# In Vitro Study of Tumor-Homing Peptide-Modified Magnetic Nanoparticles for Magnetic Hyperthermia

**DOI:** 10.3390/molecules29112632

**Published:** 2024-06-03

**Authors:** Shengli Zhou, Kaname Tsutsumiuchi, Ritsuko Imai, Yukiko Miki, Anna Kondo, Hiroshi Nakagawa, Kazunori Watanabe, Takashi Ohtsuki

**Affiliations:** 1 Department of Interdisciplinary Science and Engineering in Health Systems, Okayama University, Okayama 700-8530, Japan; p0ux6145@s.okayama-u.ac.jp (S.Z.); k_watanabe@okayama-u.ac.jp (K.W.); 2 College of Bioscience and Biotechnology, Chubu University, Aichi 487-8501, Japan; tsutsu@fsc.chubu.ac.jp (K.T.); r-imai@isc.chubu.ac.jp (R.I.); fr19115-4614@sti.chubu.ac.jp (Y.M.); fr19055-2884@sti.chubu.ac.jp (A.K.); hnakagaw@isc.chubu.ac.jp (H.N.)

**Keywords:** tumor-homing peptide, magnetic hyperthermia, magnetic nanoparticles, ferroptosis, tumor-specific delivery

## Abstract

Cancer cells have higher heat sensitivity compared to normal cells; therefore, hyperthermia is a promising approach for cancer therapy because of its ability to selectively kill cancer cells by heating them. However, the specific and rapid heating of tumor tissues remains challenging. This study investigated the potential of magnetic nanoparticles (MNPs) modified with tumor-homing peptides (THPs), specifically PL1 and PL3, for tumor-specific magnetic hyperthermia therapy. The synthesis of THP-modified MNPs involved the attachment of PL1 and PL3 peptides to the surface of the MNPs, which facilitated enhanced tumor cell binding and internalization. Cell specificity studies revealed an increased uptake of PL1- and PL3-MNPs by tumor cells compared to unmodified MNPs, indicating their potential for targeted delivery. In vitro hyperthermia experiments demonstrated the efficacy of PL3-MNPs in inducing tumor cell death when exposed to an alternating magnetic field (AMF). Even without exposure to an AMF, an additional ferroptotic pathway was suggested to be mediated by the nanoparticles. Thus, this study suggests that THP-modified MNPs, particularly PL3-MNPs, hold promise as a targeted approach for tumor-specific magnetic hyperthermia therapy.

## 1. Introduction

Hyperthermia is a low-invasive cancer therapeutic approach that kills cancer cells by heating up the affected area (41–43 °C) for more than 1 h in clinical therapy [1]. The heat sensitivity of cancer cells is higher than that of normal cells [2,3]. The effectiveness of hyperthermia has been reported in combination with other cancer therapies such as chemotherapy, radiotherapy, and immunotherapy [4,5]. These combination methods lead to side effects caused by drugs, radiation, and immunotherapy that may induce autoimmunity and nonspecific inflammation [6]; therefore, it is desirable to establish hyperthermia as an effective stand-alone treatment. However, a limitation with respect to the use of hyperthermia alone is the difficulty in rapidly raising the temperature of the target tissue while distinguishing it from the surrounding normal tissues [7]. Therefore, techniques to heat local tumor tissues within a short period of time are required.

Magnetic nanoparticles (MNPs) can play a crucial role in enabling localized heating within a short period of time [8]. Due to their biocompatibility and superparamagnetic properties, MNPs are used to induce magnetic hyperthermia by applying an alternating magnetic field (AMF) [9,10,11]. In recent years, diverse surface-modified MNPs [12] and bioconjugated MNPs with proteins and antibodies, including folate receptor, trastuzumab, and immunoglobulin G [13], have been reported to improve the therapeutic performance of MNPs during hyperthermia. The tumor-specific delivery of MNPs is expected to provide faster and improved localized heating compared to the traditional method. MNPs have been utilized in tumor-specific drug delivery [14,15,16] and in magnetic resonance imaging to detect tumors [17] and diseases such as Alzheimer’s [18]. However, methods for delivering MNPs to tumor tissues need to be further investigated. When administered intravenously, MNPs are selectively engulfed by the reticuloendothelial system, resulting in the attenuation of MNP delivery to the target tumor tissue [19]. Large MNPs (>200 nm) are recognized by the immune system and delivered to the liver and spleen [20,21], and very small MNPs (<5.5 nm) are excreted through the kidneys [20,22]. To improve the therapeutic effect of magnetic hyperthermia, it is necessary to develop highly specific delivery techniques that ensure the accumulation and sufficient residence time of MNPs in tumor tissue and cause minimal damage to surrounding healthy tissues [9,10,23].

In this study, we attempted the tumor-specific delivery of MNPs bearing tumor-homing peptides (THPs), PL1 and PL3, for inducing magnetic hyperthermia. THPs can enhance the internalization of nanoparticles via direct interactions with receptors overexpressed on the tumor cell surface or with proteins in the tumor extracellular matrix [24,25]. Thus, THPs not only have cell internalization abilities similar to those of cell-penetrating peptides (CPPs) but also have tumor-targeting abilities. For example, CREKA (amino acid sequence: CREKA) selectively binds to the fibrin–fibronectin complex in the tumor stroma, and tumor delivery and magnetic hyperthermia have been demonstrated using CREKA-conjugated MNPs [26]. Although not a study of hyperthermia, there are more examples of studies involving THPs and MNPs, including iRGD (amino acid sequence: CRGD[K/R]GP[D/E]C) that specifically targets αv integrins highly expressed in tumor vasculature, and it enabled the delivery of MNPs and magnetic resonance imaging of the target tumors [27]. An F3 peptide (amino acid sequence: KDEPQRRSARLSAKPAPPKPEPKPKKAPAKK) targets tumor blood vessels, and F3-conjugated polymer nanoparticles encapsulating photodynamic therapy agents and imaging agents (iron oxide) were demonstrated to be delivered to the target site [28]. A tumor-homing peptide CGKRK (the name represents its sequence) was also reported to be conjugated with a proapoptotic peptide and then associated with iron oxide nanoparticles, which enabled the apoptosis of the target cells and imaging of brain tumors [29]. Engineered extracellular vesicles (EVs) with MNPs and tumor-homing RGD peptides (amino acid sequence: RGD) allowed for enhancement in autologous EVs delivering paclitaxel in pancreatic cancer, but no additional therapeutic effect of the permanent magnet of MNPs was observed. [30].

PL1 (amino acid sequence: PPRRGLIKLKTS) and PL3 (amino acid sequence: AGRGRLVR) have dual targets and can deliver nanoparticles to solid tumors [31,32]. PL1 can specifically bind to the C-isoform of tenascin-C (TNC-C) and fibronectin extra domain B (FN-EDB), which are overexpressed in many solid tumors [33,34,35]. PL3 targets TNC-C, and neuropilin-1 (NPR-1) is upregulated in most solid tumors [32,36]. Their interaction was characterized by fluorescent anisotropy experiments: PL1 and FN-EDB (K_D_ = 1.11 ± 0.27 μM), PL1 and TNC-C (K_D_ = 1.14 ± 0.39 μM), PL3 and NPR-1 (K_D_ = 1.1 ± 0.2 μM), and PL3 and TNC-C (K_D_ = 51 ± 19 μM) [32,37]. The excellent targeting of PL1 and PL3 to tumor tissues is expected to enhance the efficiency of tumor cell penetration and local heating by modifying the surface of MNPs. In addition, the dual targeting ability of these THPs may help MNPs distribute evenly over the target tissue, which is necessary for the uniform heating of the tissue. However, the applications of PL1 and PL3 in magnetic hyperthermia have not yet been explored. In this study, we prepared PL1- and PL3-modified MNPs using nanoflower-shaped dextran-coated MNPs, evaluated their particle size, cell specificity, and intracellular uptake, and attempted to induce magnetic hyperthermia.

## 2. Results and Discussion

### 2.1. Syntheses of THP-MNPs

**Figure 1:**
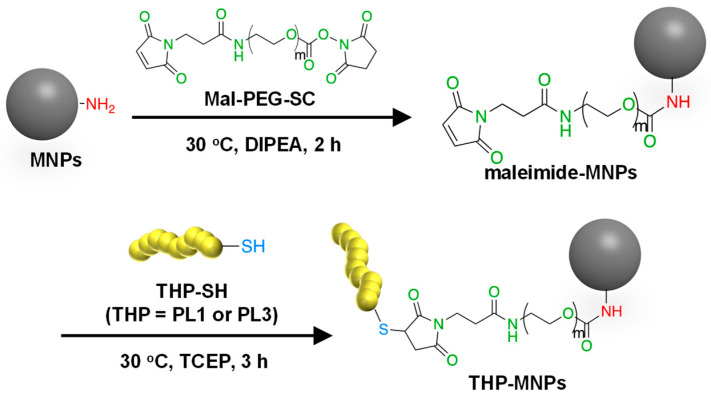
A scheme for the synthesis of THP-modified MNPs.

The flower-shaped aminated 50 nm Synomag^®^-D50 consisting of iron oxide in a matrix of 40 kDa dextran was used as MNPs because flower-shaped MNPs have superior efficiency in inducing a temperature rise and intracellular uptake and are less prone to aggregation [38]. Among commercial superparamagnetic iron oxide nanoparticles, Synomag^®^-D50 showed relatively high rates of specific absorption and high heating efficiency [39]. To improve the specific binding and invasive ability of the MNPs to the tumor cells, two types of tumor-homing peptides were attached to the surface of the MNPs as described in Materials and Methods (Figure 1). PL1 and PL3 contain amino acids with side-chain NH_2_ groups such as arginine and lysine. These THPs can bind to both the maleimide (Mal) and succinimidyl ester (SC) groups of a crosslinker Mal-PEG-SC. Thus, Mal-PEG-SC first reacted with the aminated MNPs at the SC group, and the product (maleimide–MNPs) was then allowed to react with THP. Maleimide–MNPs were analyzed using Fourier transform infrared spectroscopy (Figure S1), confirming the successful synthesis of maleimide-modified MNPs. After THP-MNP preparation, the absorbance at 400 nm, where there is no absorbance of the THPs, was measured to determine the concentration of MNPs (Figure S2). The recovery rates obtained for the MNPs after the entire procedure were 57.3% for PL1-MNPs and 39.6% for PL3-MNPs. At MNP concentrations of 800 μg/mL or lower, THP-MNP was well dispersed in water even without sonication. The modification of the maleimide–MNPs with THPs was confirmed using the bicinchoninic acid (BCA) assay for peptide–protein quantitation (Figure S3). The BCA absorption peaks at 562 nm of the modified and unmodified MNPs at the same MNP concentration (100 μg/mL) were compared. The absorption peaks obtained for the unmodified MNPs and maleimide–MNPs were significantly lower than that of PL1-MNPs and PL3-MNPs, indicating the successful modification of the MNPs with the THPs. Based on the results obtained from the BCA assay, 1.2 μg of PL1 and 1.7 μg of PL3 were estimated to bind to 100 μg of MNPs.

### 2.2. The Characterization of the THP-MNPs

**Figure 2:**
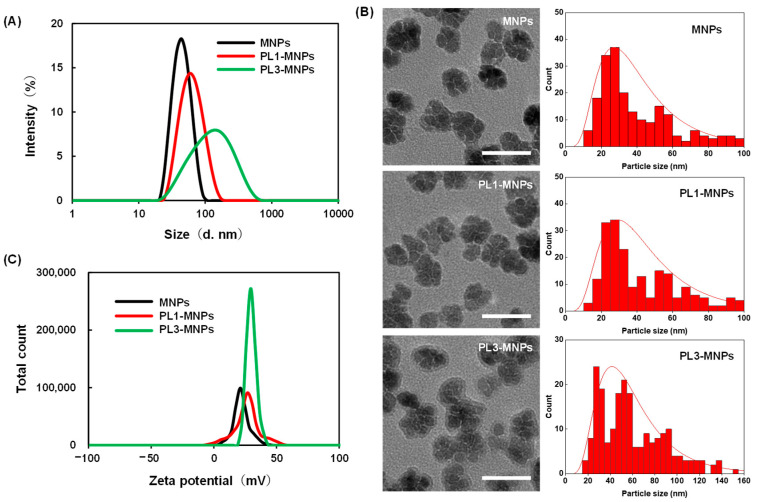
The particle size distribution measured by dynamic light scattering (**A**), transmission electron micrographs, and particle size distribution curves (**B**) and zeta potential (**C**) of unmodified MNPs, PL1-MNPs, and PL3-MNPs. Scale bars in (**B**) indicate 50 nm.

The particle sizes and zeta potentials of PL1-MNPs and PL3-MNPs after peptide modification were measured (Figure 2A). Compared to the unmodified MNPs (42.0 ± 3.3 nm, polydispersity index (PDI) = 0.08 ± 0.01), the particle size of the PL1-MNPs (57.3 ± 7.5 nm, PDI = 0.13 ± 0.01) increased 1.36-fold after surface modification with THP, while the particle size of PL3-MNPs (103 ± 29 nm, PDI = 0.28 ± 0.001) increased more than two-fold. The larger particle size of the PL3-MNPs may be due to the large number of hydrophobic amino acids at the N-terminus of PL3, which promotes aggregation. Because PL1- and PL3-MNPs showed a single particle size distribution and the average particle size did not exceed 200 nm, these THP-MNPs were expected to accumulate in tumors owing to enhanced permeability and retention (EPR) effects [40,41,42]. In the transmission electron micrographs, PL1 and PL3 did not significantly alter the nanoflower shape of the individual MNPs, but modification with PL3 appeared to slightly promote aggregation between MNPs (Figure 2B, left). The particle size distribution curves analyzed using transmission electron micrographs (Figure 2B, right) show that PL3-MNPs included larger particles (60.7 ± 30.8 nm) than those of MNPs (41.7 ± 25.3 nm) and PL1-MNPs (45.3 ± 25.6 nm), which were almost consistent with the results of dynamic light scattering. The zeta potential of PL1-MNPs (24.7 ± 0.8) and PL3-MNPs (29.6 ± 0.2) also increased compared to the unmodified MNPs (22.5 ± 0.2 mV), which may facilitate their binding to the anionic surface of the cells (Figure 2C).

### 2.3. Cell Specificity of THP-Modified MNPs

**Figure 3:**
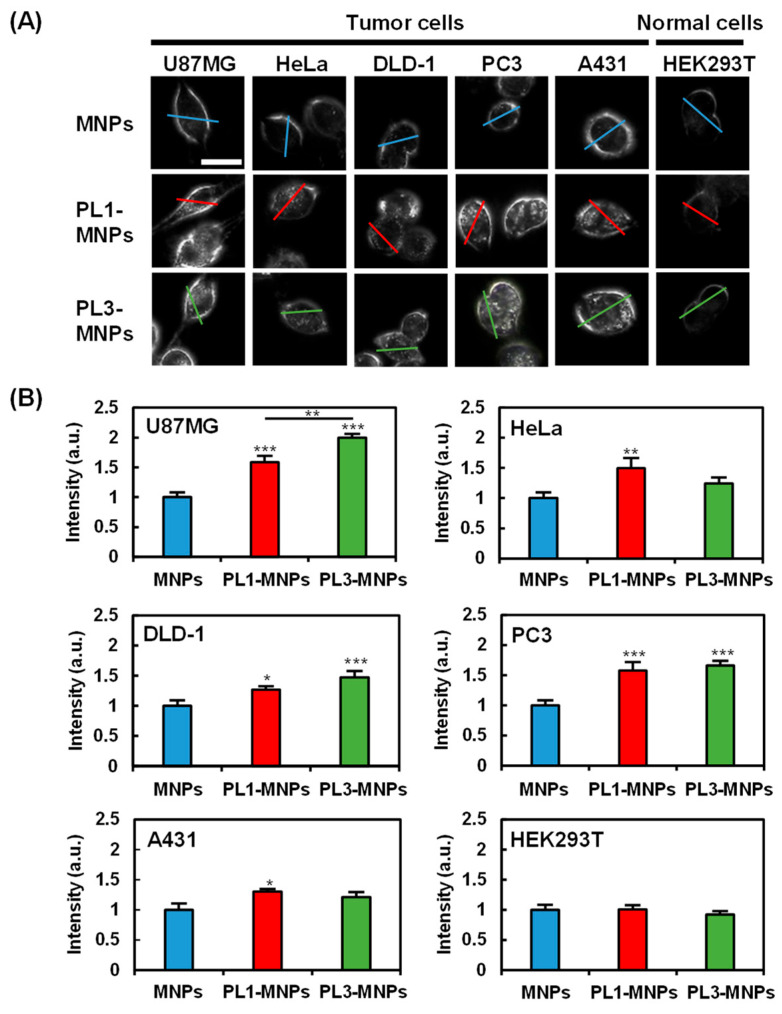
Cell specificity of THP-modified MNPs. (**A**) Dark-field images of tumor cells (U87MG, HeLa, DLD-1, PC3, and A431) and normal cells (HEK293T) treated with unmodified MNPs, PL1-MNPs, or PL3-MNPs with MNP concentrations of 50 μg/mL. Line scanning profiles of MNPs (blue line), PL1-MNPs (red line), and PL3-MNPs (green line) on images are shown in Figure S4. Scale bars indicate 20 μm. (**B**) Histograms of line scanning intensities normalized with MNPs. Data represent mean ± standard errors of mean (SEMs) of twenty-one independent experiments. *p* values were determined by one-way analysis of variance; *** *p* < 0.001, ** *p* < 0.01, * *p* < 0.05. Data points marked with ***, **, and * are statistically different from data point of same cell lines without THP-modified MNPs.

To provide specificity comparison among unmodified MNPs, PL1-MNPs, and PL3-MNPs, low MNP concentrations (50 μg/mL) of the particles were exposed to five different cancer cell lines (U87MG, HeLa, DLD-1, PC3, and A431 cells), as well as one normal cell line (HEK293T cells) for 1 h. The cellular uptake of the MNPs was imaged using dark-field microscopy (Figure 3). The light intensities along the lines traversing the cells were considered to reflect the uptake of the MNPs within the cells (Figure 3A and Figure S4). The average intensity values demonstrated that, compared to MNPs, THP-modified PL1-MNPs and PL3-MNPs exhibited increased specificity for different cells (Figure 3B). Due to the nature of dark-field microscopy, the light intensity detected comes from the light scattering of the observed objects, not only from the nanoparticles but also from the cell membrane. Although it appears from the histogram (Figure 3B) that large numbers of all type of MNPs penetrated normal (HEK293T) cells, nanoparticle-shaped signals were hardly observed in normal cells (Figure 3A). Unlike unmodified MNPs, PL1-MNPs showed a moderate increase in uptake in U87MG (1.6-fold), PC3 (1.6-fold), and HeLa (1.5-fold) cells, a slight increase in DLD-1 and A431 cells, and no significant change in uptake in HEK293T cells (Figure 3B). PL3-MNPs, in comparison to unmodified MNPs, displayed a significantly higher uptake in U87MG (2.0-fold) cells, a moderate increase in PC3 (1.7-fold) and DLD-1 (1.5-fold) cells, a slight increase in HeLa and A431 cells, and no significant change in uptake in HEK293T cells. Thus, these results highlight that THP-modified MNPs have a stronger tendency to adhere and to penetrate various tumor cells than normal cells (Figure 3B). In particular, the specificity of PL3-MNPs for U87MG cells was high. Thus, we decided to use these combinations in the subsequent experiments.

### 2.4. Intracellular Iron Uptake

**Figure 4:**
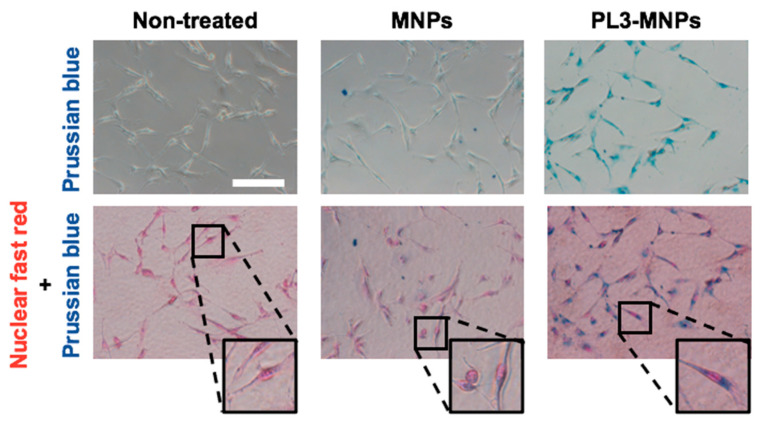
Intracellular uptake study of MNPs and PL3-MNPs in U87MG cells by Prussian blue staining. Phase contrast images with iron oxide nanoparticles stained with Prussian blue and cell nucleus stained with nuclear fast red. Scale bar indicates 100 μm.

Figure 3 shows MNPs binding to or entering the cell but does not show that they have entered the cell. To obtain clear images of the cellular uptake of MNPs and PL3-MNPs into the U87MG cells, the cells were examined using an iron staining kit. The images of cells after iron staining clearly demonstrated a significantly higher quantity of PL3-MNPs taken up by U87MG cells compared to the unmodified MNPs (Figure 4). Furthermore, the internalized PL3-MNPs accumulated within the cytoplasm with no observable presence in the cell nucleus.

### 2.5. In Vitro Hyperthermia

**Figure 5:**
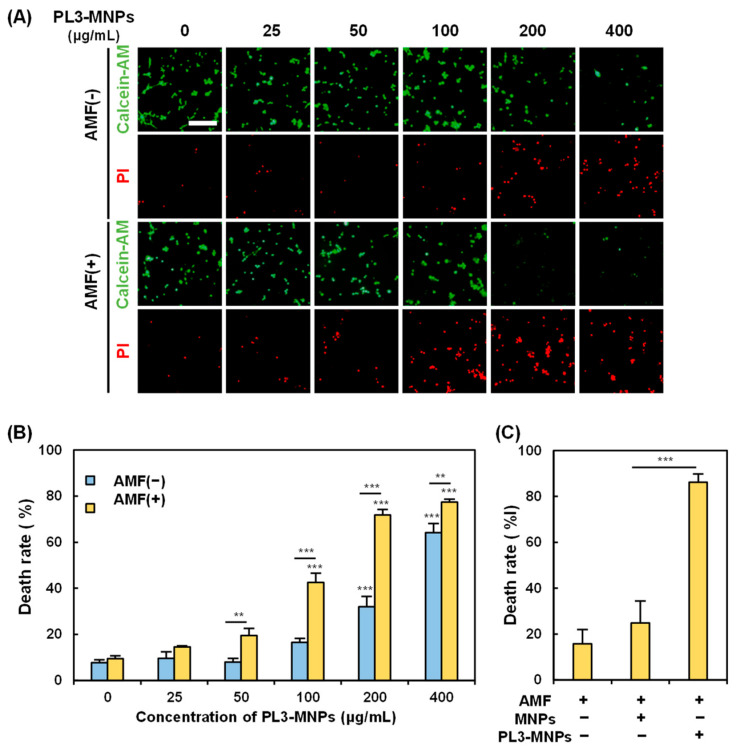
Assessment of PL3-MNP therapeutic performance. (**A**) Calcein-AM (living cells) and PI (dead cells) images of U87MG with and without AMF irradiation after treatment with PL3-MNPs. Scale bar indicates 100 μm. (**B**,**C**) Hyperthermia effect evaluated by cell death rate. (**B**) Data represent mean ± SEM of three independent experiments. *p* values were determined by two-way analysis and Bonferroni’s multiple comparisons test; *** *p* < 0.001, ** *p* < 0.01. Data points marked with *** are statistically different from data point without PL3-MNPs. (**C**) Cells were treated with unmodified MNPs or PL3-MNPs with MNP concentrations of 200 μg/mL. Data represent mean ± SEM of three independent experiments. *p* values were determined by one-way analysis of variance; *** *p* < 0.001.

The in vitro magnetic hyperthermia performance of the PL3-MNPs was examined by treating the U87MG cells with PL3-MNPs in an AMF. The death rate of U87MG cells was measured 24 h after exposure to the AMF. In the absence of the AMF, almost no U87MG cell death was observed when the concentration of PL3-MNPs was 50 μg/mL or less (Figure 5A). The concentrations used here were weight concentrations of the MNPs (Synomag^®^-D50), not those including THPs. When the concentration of PL3-MNPs was 100 μg/mL and in the absence of the AMF, the number of dead cells did not significantly increase. However, upon AMF exposure, dead cells were clearly observed at concentrations above 100 μg/mL (Figure 5A). The death rate of U87MG cells was calculated by counting the number of live and dead cells after double staining with calcein-AM and propidium iodide (PI). The death rate with PL3-MNPs at 100 μg/mL was 16.5% in the absence of the AMF, whereas it was 42.5% in the presence of the AMF, 2.6 times higher than the AMF (−) condition (Figure 5B). The therapeutic effect was significant at a concentration above 100 μg/mL with respect to PL3-MNPs and was maximal (death rate > 70%) at a concentration above 200 μg/mL with respect to PL3-MNPs. When 400 μg/mL of PL3-MNPs was used, the death rates did not change significantly when compared to the use of 200 μg/mL PL3-MNPs in the absence of the AMF. The concentration dependence correlates with the irradiation time-dependent temperature increase; as when the PL3-MNP concentration exceeds 200 μg/mL, the temperature suitable for hyperthermia (43 °C) is also reached after 15 min (Figure S5). In contrast to PL3-MNPs, MNPs at a concentration of 200 μg/mL did not significantly induce cell death even in the presence of the AMF (Figure 5C). An approximately 4-fold difference in the therapeutic effect was observed between MNPs and PL3-MNPs.

### 2.6. Ferroptosis with PL3-MNPs

**Figure 6:**
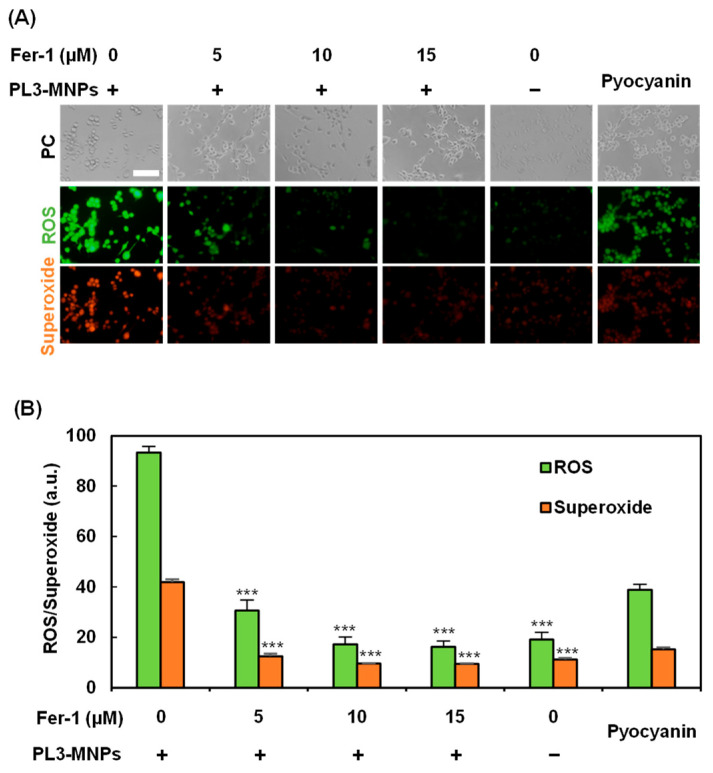
Verification of ferroptosis inducibility of PL3-MNPs. (**A**) Phase contrast (PC), reactive oxygen species (ROS), and superoxide images of U87MG cells treated with PL3-MNPs (200 μg/mL MNP concentration) and Fer-1. Pyocyanin (1 mM) was used as ROS-generating positive control. Scale bar indicates 100 μm. (**B**) Production of ROS and superoxide. Data represent mean ± SEM of three independent experiments. *p* values were determined by two-way ANOVA and Tukey post honestly significant difference test; *** *p* < 0.001. Data points marked with *** are statistically different from data point with PL3-MNPs without Fer-1.

Treatment with PL3-MNPs at concentrations above 200 μg/mL resulted in cell death, even without exposure to the AMF (Figure 5B). Therefore, cell death can be induced via pathways involving the particles themselves. Recently, iron oxide nanoparticles have been reported to induce cell death via the ferroptosis pathway [43,44]. This is because excess ferrous ions in iron oxide particles react with unsaturated fatty acids that are highly expressed on the cell membrane, causing peroxidation and ultimately inducing cell death due to changes in cell membrane permeability. To determine whether ferroptosis can be induced by 200 μg/mL PL3-MNPs without exposure to the AMF, the effect of the ferroptosis inhibitor Ferrostatin-1 (Fer-1) was examined. Ferroptosis was evaluated by the production of reactive oxygen species (ROS) and superoxide, which were visualized using a Total ROS/superoxide detection kit (Figure 6). Compared to U87MG cells that had not been treated with Fer-1, the production of ROS and superoxide in these cells significantly reduced after pretreatment with Fer-1. When the concentration of Fer-1 was above 10 μM, the production of ROS and superoxide with PL3-MNPs was the same as without PL3-MNPs. (Figure 6B). These results indicate that PL3-MNPs induce cell death via the ferroptotic pathway. We confirmed the correlation between Fer-1 concentration and cell death in the presence of PL3-MNPs. The death rate of U87MG cells treated with PL3-MNPs (31.8%) significantly decreased in the presence of Fer-1 (1.6%), indicating that the cell death by PL3-MNPs without the AMF occurred via the ferroptosis pathway (Figure S6).

## 3. Materials and Methods

### 3.1. Synthesis of THP-Modified MNPs

The aminated MNPs, 50 nm Synomag^®^-D50, which consist of about 55% (*w*/*w*) iron oxide in a matrix of 40 kDa dextran, were purchased from Micromod Partikeltechnologie GmbH (Rostock, Germany). The MNPs were modified with maleimide using Mal-PEG-SC, molecular weight 2 K (Biopharma PEG Scientific, Watertown, MA, USA). Tumor-homing peptides (THPs) were coupled to the MNPs via a thioether bond between the thiol groups added to the terminal cysteine residues of THPs (Figure 1). For the THPs, we used PL1 (*N*-PPRRGLIKLKTSWGC-*C*; the C-terminal Cys was added for the reaction with maleimide) and PL3 (*N*-CLAWAGRGRLVR-*C*; the N-terminal Cys was added for the reaction with maleimide). Briefly, MNPs (500 μg) and Mal-PEG-SC (500 μg) were mixed in 1 mL of DMSO containing 5% *N*,*N*-diisopropylethylamine (DIPEA) with continuous shaking at 30 °C for 2 h. It was then allowed to react with maleimide–MNPs (250 μg) and THP (5 μg) in phosphate-buffered saline (PBS) containing 60 nM tris(2-carboxyethyl)phosphine (TCEP) with continuous shaking at 30 °C for 3 h (Figure 1). TCEP was used to prevent the formation of unwanted disulfide bonds between THP molecules containing Cys. Using a 100 kDa Microsep Advance Centrifugal Device (Pall Corporation, New York, NY, USA), the products (both maleimide–MNPs and THP-MNPs) were purified, and the solvent was replaced by pure water.

### 3.2. Characterization

The MNP weight concentrations (without the weight of THP moiety) of MNPs and THP-modified MNPs were determined by measuring the absorbance of the MNPs at 400 nm using a NanoPhotometer (C40, Implen GmbH, Munich, Germany). A Zetasizer Pro (Malvern Instruments, Malvern, UK) was used to assess the zeta potentials and particle sizes of the THP-MNPs. The zeta potentials were measured using 10 μg/mL THP-MNPs dissolved in milliQ water at 25 °C. Transmission electron micrographs of the THP-MNPs were observed using an H-7650 transmission electron microscope (Hitachi, Ibaragi, Japan). The particle size distribution in the micrographs was analyzed using the open source software ImageJ (version 1.53a), originally developed by the National Institutes of Health [45,46]. The particle size distributions were measured using 200 μg/mL THP-MNPs dissolved in T buffer (20 mM 4-(2-hydroxyethyl)-1-piperazineethanesulfonic acid (HEPES)-KOH [pH 7.6], 115 mM NaCl, 5.4 mM KCl, 1.8 mM CaCl_2_, 0.8 mM MgCl_2_, and 13.8 mM glucose) at 25 °C. To quantify the peptides conjugated to MNPs, a BCA protein assay (Thermo Fisher Scientific, Tokyo, Japan) was performed.

### 3.3. Cell Culture

All cell lines were cultured at 37 °C in 5% CO_2_ atmosphere, unless otherwise indicated. U87MG (human glioblastoma) and A431 (human squamous carcinoma) cells were cultured in E-MEM medium (Fujifilm, Tokyo, Japan) with 10% fetal bovine serum (FBS; Sigma-Aldrich, St. Louis, MI, USA), 1% of 100 × nonessential amino acid solution (Fujifilm, Tokyo, Japan), 1 mM sodium pyruvate (Thermo Fisher Scientific, Tokyo, Japan), 100 U/mL penicillin, and 100 μg/mL streptomycin (Gibco, Emeryville, CA, USA). Penicillin and streptomycin were used to prevent bacterial contamination. HeLa (human cervical carcinoma), PC3 (human prostate carcinoma), and DLD-1 (human colon adenocarcinoma) cells were cultured in RPMI1640 medium (Nacalai Tesque, Kyoto, Japan) containing 10% FBS and 100 U/mL penicillin and 100 μg/mL streptomycin. HEK-293T (human embryonic kidney) cells were cultured in high-glucose D-MEM medium (Fujifilm, Tokyo, Japan) containing 10% FBS, 100 U/mL penicillin, and 100 μg/mL streptomycin.

### 3.4. Evaluation of Cell Specificity of THP-Modified MNPs

U87MG, A431, HeLa, PC3, DLD-1, and HEK293T cells were seeded with 2 mL of E-MEM or RPMI1640 medium in a 12-well plate with a 1.8 cm cover glass (2 × 10^5^ cells/well). After 24 h of incubation, the cover glasses were transferred to 3.5 cm dishes, and 100 μL of 50 μg/mL MNP concentrations of MNP, PL1-MNP, or PL3-MNP solution in T buffer (20 mM HEPES-KOH [pH 7.6], 115 mM NaCl, 5.4 mM KCl, 1.8 mM CaCl_2_, 0.8 mM MgCl_2_, and 13.8 mM glucose) was added. After incubation at 37 °C for 1 h, it was washed 3 times with 1 × PBS and treated with etching solution (1 × PBS containing 1 mM of K_3_Fe(CN)_6_ and Na_2_S_2_O_3_) for 3 min to remove MNPs that were not uptaken into the cells. Cells treated with MNPs were observed by dark-field microscopy using a CX43 microscope (Olympus, Tokyo, Japan). The cell specificity of MNPs, PL1-MNPs, and PL3-MNPs was evaluated by measuring the average light intensity without the cell membrane using ImageJ software [45]. MNPs not modified by THP were used as controls to evaluate the ability of PL1-MNPs and PL3-MNPs to invade different cell types.

### 3.5. Intracellular Iron Uptake

The uptake and intracellular distribution of MNPs and PL3-MNPs in U87MG cells were investigated using an Iron Stain Kit (ScyTek Laboratories, Logan, UT, USA). Briefly, 6 × 10^3^ U87MG cells were seeded in each well of an 18-well microchamber slide (ibidi GmbH, Graefelfing, Germany). After 48 h of culture, the cells were washed with T buffer and treated with 100 μL of a 200 μg/mL MNP concentration of PL3-MNPs dissolved in T buffer. After treatment for 24 h, the cells were fixed with 4% paraformaldehyde (Nacalai Tesque, Kyoto, Japan) for 20 min. The cells were then stained with Prussian blue solution at 37 °C for 30 min and double-stained with nuclear fast red for 1 min. Finally, the cells were washed thrice with 1 × PBS and observed under a confocal microscope.

### 3.6. In Vitro Hyperthermia Assessment

For standard cell hyperthermia experiments, U87MG cells were first seeded in an 18-well microchamber slide at a density of 6 × 10^3^ cells/well with 100 μL E-MEM medium, incubated for 48 h at 37 °C and 5% CO_2_ atmosphere. After washing once with 100 μL T buffer, different MNP concentrations (0–400 μg/mL) of PL3-MNPs along with 100 μL T buffer were added to the plate, and the cells were cultured for another 24 h. The cells were then washed twice with E-MEM medium containing 10% fetal bovine serum, 1% 100X nonessential amino acid solution, 1 mM sodium pyruvate, 100 U/mL penicillin, and 100 μg/mL streptomycin. A high-frequency alternating magnetic field (AMF) system (Dai-ichi High Frequency Co. Ltd., Kanagawa, Japan) was applied with *F* = 105 kHz and *H* = 22.1 kA/m for 30 min. The cells were then incubated for 24 h at 37 °C and 5% CO_2_ atmosphere and double-stained with calcein-AM (Dojindo, Kumamoto, Japan) and propidium iodide (PI) (Molecular Probes, Eugene, OR, USA) to visualize cell death caused by the different treatments.

### 3.7. Detection of Ferroptosis

To evaluate the ferroptosis inducibility of PL3-MNPs, U87MG cells were seeded in an 18-well microchamber slide at a density of 6 × 10^3^ cells/well with 100 μL E-MEM medium and incubated for 48 h at 37 °C and 5% CO_2_ atmosphere. After washing once with 100 μL T buffer, U87MG cells were pretreated with different concentrations (0–20 μM) of Fer-1 (Cayman Chemical, Ann Arbor, MI, USA) dissolved in 1 × PBS for 3 h at 37 °C. The cells were then treated with 200 μg/mL PL3-MNPs dissolved in 100 μL T buffer for 2 h at 37 °C. The cells were then washed with E-MEM medium and incubated for another 24 h at 37 °C. The generated reactive oxygen species were visualized using a Total ROS/Superoxide Detection Kit (Enzo Life Sciences, New York, NY, USA) and observed under a fluorescence microscope.

To observe ferroptosis induced by PL3-MNPs, the effect of the ferroptosis inhibitor Fer-1 on cell death rate was evaluated. In an 18-well microchamber slide, 6 × 10^3^ U87MG cells were seeded in each well. After 48 h of incubation at 37 °C, the cells were washed with T buffer and pretreated with 10 μM Fer-1 and 1 × PBS for 3 h at 37 °C. The cells were washed with T buffer and treated with 200 μg/mL MNP concentration of PL3-MNPs for 24 h at 37 °C. The cells were then double-stained with calcein-AM and PI to visualize live and dead cells.

### 3.8. Statistical Analysis

All values are presented as the standard error of the mean (SEM) of at least three independent experiments. Statistical analysis was performed using EZR software (version 1.60) [47]. Statistical significance was set at *p* < 0.05.

## 4. Conclusions

This study successfully developed and characterized MNPs modified with THPs, PL1, and PL3. The THP-modified MNPs, in particular PL3-MNP, were shown to be promising candidates for tumor-specific magnetic hyperthermia. Through the strategic use of THPs such as PL3, MNPs exhibit specific binding and internalization into tumor cells, thereby enhancing their therapeutic potential. The in vitro investigations established the increased cell specificity, intracellular uptake, and magnetic hyperthermia performance of the PL3-MNPs. Cell death was strongly induced when PL3-MNPs were administered and exposed to the AMF; however, cell death was induced even without exposure to the AMF (38% cell death compared to AMF(+)) when PL3-MNPs were administered at 200 μg/mL. Cell death without AMF exposure induces PL3-MNP-mediated ferroptosis. This study suggests that MNPs modified with the tumor-homing peptide PL3 have therapeutic effects by delivering MNPs specifically to cancer cells and inducing cell death through hyperthermia and ferroptosis.

We previously investigated the mechanism of heat stress response in cancer cells and found that thermal sensitizers can enhance the cell-killing effect of heat stress by inhibiting the formation of stress-induced nuclear granules to downregulate the expression of heat shock proteins [48]. In the future, the modification of THP-MNPs with drug molecules, such as thermal sensitizers and proapoptotic peptides/miRNAs [49,50], is expected to improve cancer treatment efficiency. This study anticipates further progress towards in vivo studies to validate the therapeutic effects of THP-MNPs on magnetic hyperthermia.

## Data Availability

The data that support the findings of this study are available on request from the corresponding author.

## Supplementary Materials

The following supporting information can be downloaded at https://www.mdpi.com/article/10.3390/molecules29112632/s1, Figure S1: Fourier transform infrared spectroscopy (FT-IR) spectra; Figure S2: Quantification of MNPs, maleimide–MNPs, and THP-modified MNPs; Figure S3: THP attachment to MNPs shown by BCA assay; Figure S4: Line scan profiles of THP-MNPs in dark-field cell images; Figure S5: AMF exposure time-dependent temperature increase in the presence of PL3-MNPs; Figure S6: Verification of ferroptosis inducibility of PL3-MNPs.
