# Supplementary material for: In Vitro Study of Tumor-Homing Peptide-Modified Magnetic Nanoparticles for Magnetic Hyperthermia
Source: Molecules. 2024 Jun 3;29(11):2632. doi: 10.3390/molecules29112632 (PMC11174109; doi:10.3390/molecules29112632)
Supplement: Supplementary file 1 [file molecules-29-02632-s001.zip › molecules-3016331-supplementary.pdf]

Supplemental Materials for "*In vitro* study of tumor-homing peptide-modified magnetic nanoparticles for magnetic hyperthermia"

Shengli Zhou [1], Kaname Tsutsumiuchi [2], Ritsuko Imai [2], Yukiko Miki [2], Anna Kondo [2], Hiroshi Nakagawa [2], Kazunori Watanabe [1] and Takashi Ohtsuki [1,*]

[1]Department of Interdisciplinary Science and Engineering in Health Systems, Okayama University, Okayama 700-8530, Japan
[2] College of Bioscience and Biotechnology, Chubu University, 1200 Matsumoto, Kasugai 487-8501, Japan

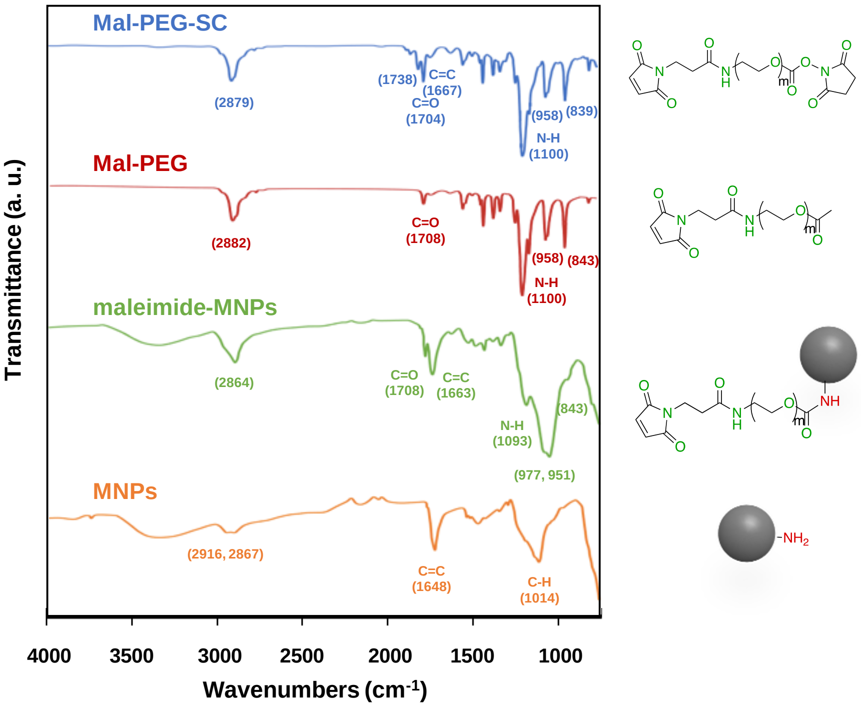

Figure S1. Fourier transform infrared spectroscopy (FT-IR) spectra. The reaction of MNPs with Mal-PEG-SC was confirmed by FT-IR (5500a, Agilent Technologies, California, USA). The peaks at 1093 and 1708 cm^{-1} corresponding to N-H and C=O bonds, respectively, were observed on maleimide-MNPs. In addition, multiple peaks in the range of 650–2000 cm^{-1} in the spectra of maleimide-MNPs and Mal-PEG were almost identical to each other, confirming the successful synthesis of maleimide-MNPs.

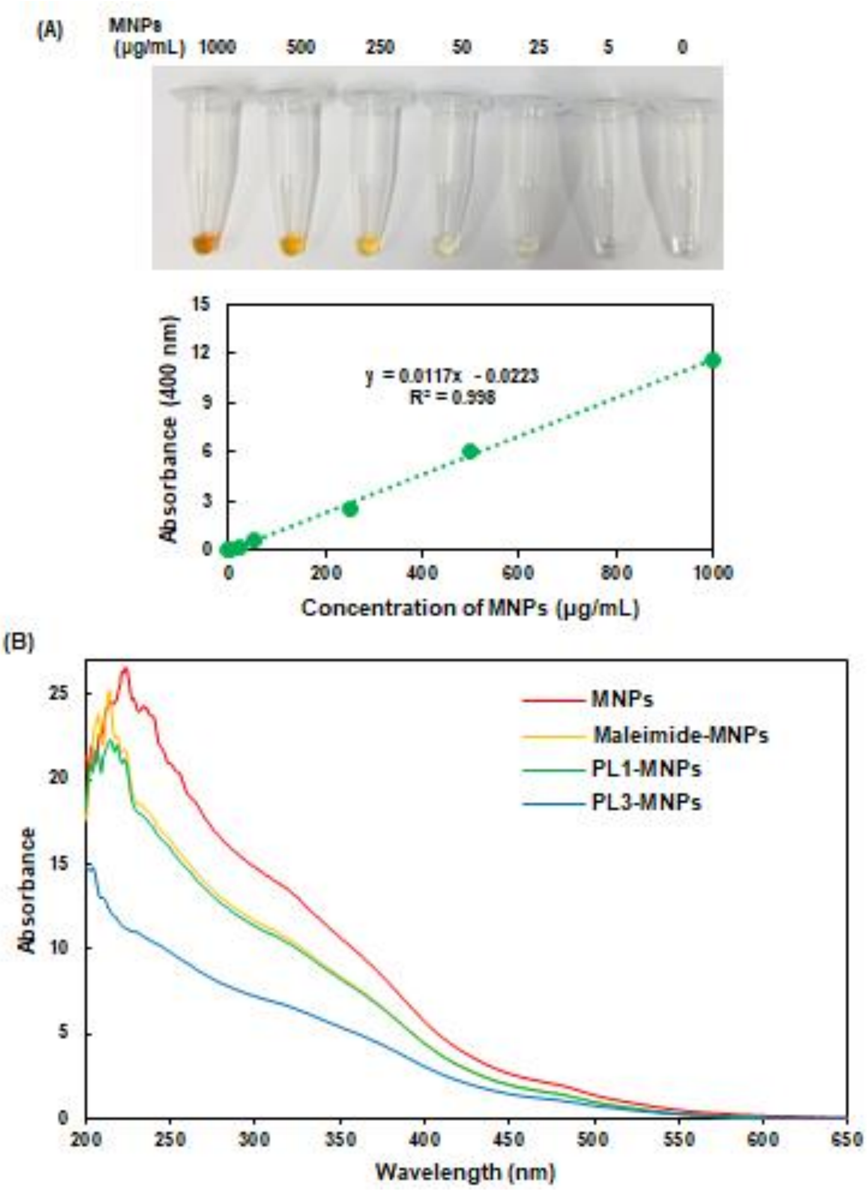

Figure S2. Quantification of MNP concentrations in the solutions of MNPs, maleimide-MNPs, and THP-modified MNPs. (A) Standard curve measurement using known concentrations (0, 5, 25, 50, 250, 500, 1000 μg/mL) of MNPs. (B) UV absorbance spectra of MNPs (500 μg/mL in 1 x PBS), maleimide-MNPs, PL1-MNPs, and PL3-MNPs. Maleimide-MNPs, PL1-MNPs, and PL3-MNPs were synthesized using the reaction mixture containing 500 μg/mL MNPs, purified by ultrafiltration, and then adjusted to the same volume (1 mL) as the original reaction solution containing 500 μg/mL MNPs by adding PBS (though MNPs concentration would have been reduced by purification). These solutions were used for the measurements. The spectra were measured using NanoPhotometer (Implen, Munich, Germany). MNP concentrations in PL1-MNPs and PL3-MNPs were calculated using their absorbances at 400 nm and the standard curve of MNP solution.

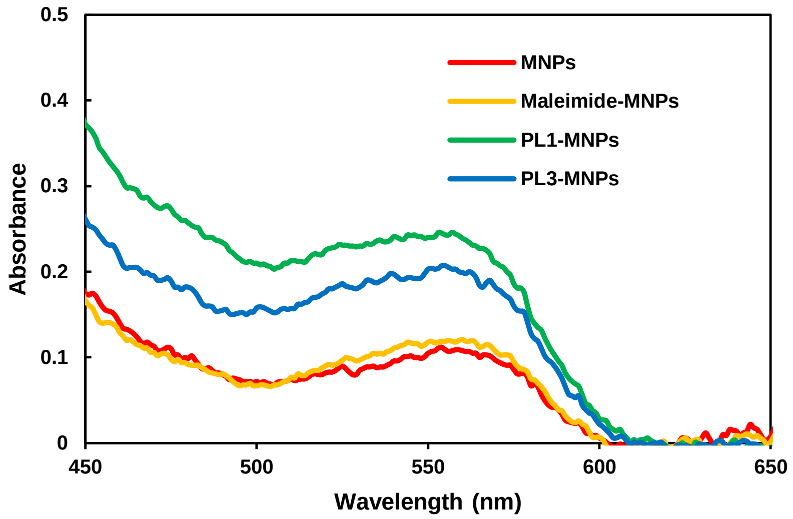

Figure S3. THP attachment to MNPs shown by BCA assay. THP-modified and unmodified MNP solutions (containing 100 μg/mL MNPs, in 1×PBS) were allowed to react with equal volume of BCA working reagent at 60 °C for 1 h, post which their absorbance were measured. Peptide concentrations in PL1-MNP and PL3-MNP solutions were calculated using standard curves plotted with known concentrations (0, 0.5, 1.0, 2.5, 5.0, 25 μg/mL) of PL1 and PL3 solutions using their absorbances at 562 nm.

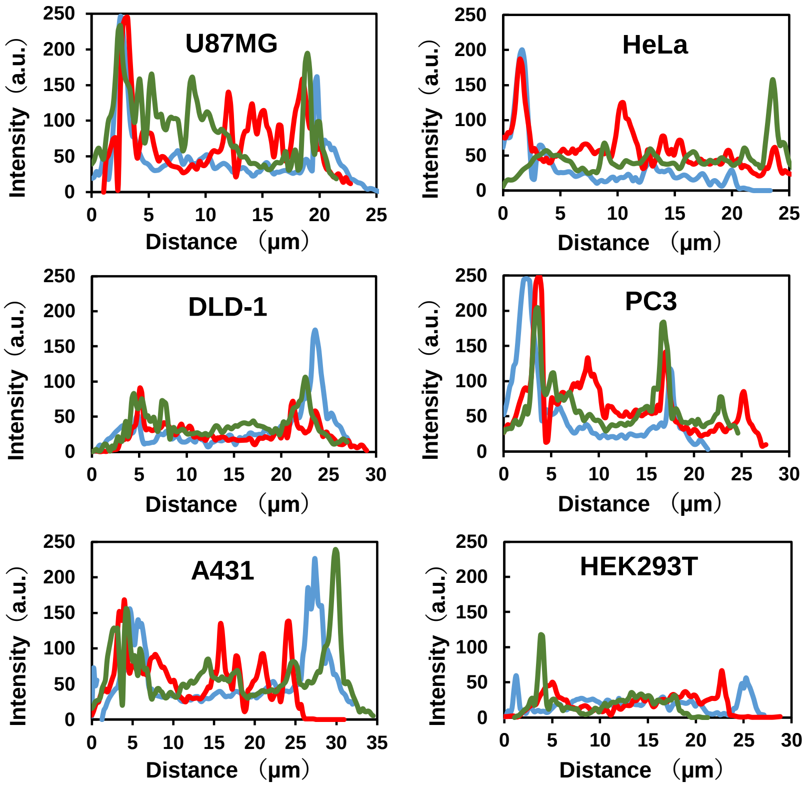

Figure S4. Line-scan profiles of THP-MNPs in dark-field cell images. The line-scan was performed on MNPs (blue line), PL1-MNPs (red line), and PL3-MNPs (green line) of Figure 3A.

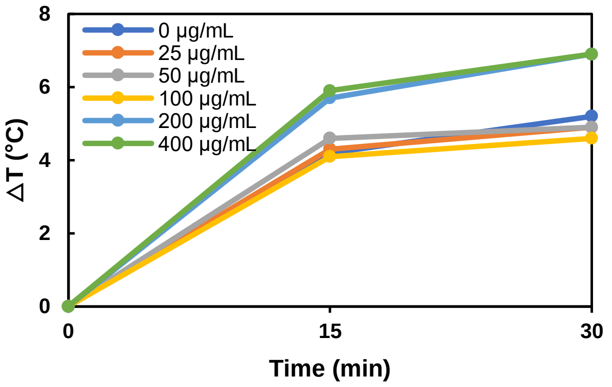

Figure S5. AMF exposure time-dependent temperature increase in the presence of PL3-MNPs. U87MG cells (6×10^3 cells/well) were seeded in an 18-well microchamber slide, incubated for 48 h, and treated with different MNP concentrations (0-400 µg/mL) of PL3-MNPs for 24 h. After the treatment, cells were washed twice with PBS (-) to remove free PL3-MNPs, and 100 µL of E-MEM medium containing 10% fetal bovine serum, 1% 100X nonessential amino acid solution, 1 mM sodium pyruvate, 100 U/mL penicillin, and 100 µg/mL streptomycin was added. The solution was then continuously exposed to AMF (F = 105 kHz, H = 22.1 kA/m) for 30 min and the temperature of the solution was measured using an optical fiber thermometer FL-2000 (Anritsu Meter Co. Ltd., Japan) with a fiber probe (plastic type, FS600-2M, Anritsu Meter Co. Ltd., Japan).

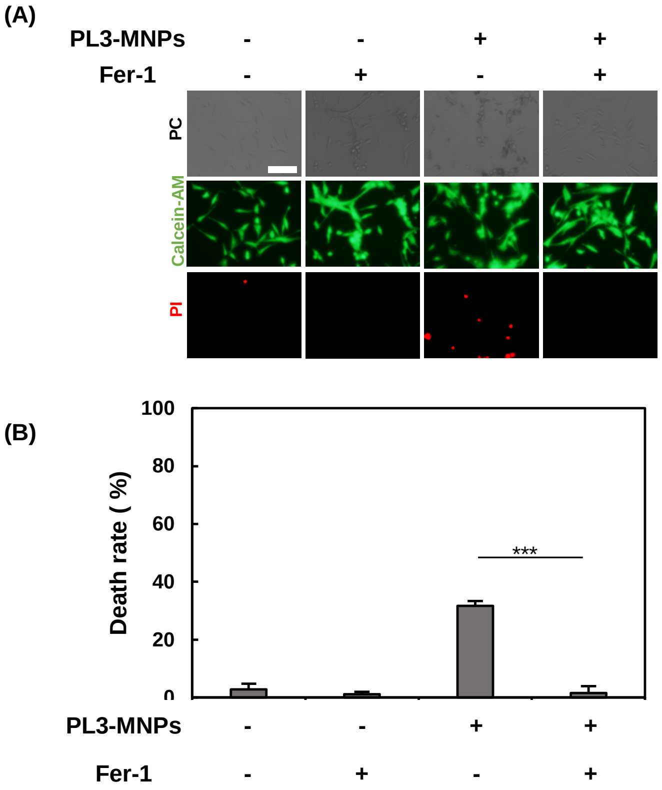

Figure S6. Verification of ferroptosis inducibility of PL3-MNPs. (A) Phase contrast (PC), Calcein-AM, and PI images of U87MG cells treated with PL3-MNPs (containing 200 μg/mL MNPs) and 10 μM Fer-1. Scale bars indicate 100 μm. (B) Death rate of U87MG cells after incubation with PL3-MNPs and Fer-1. Data represent means ± SEM of three independent experiments. The p values were calculated using two-way ANOVA and Tukey test; ***$p < 0.001$.
